# Performance Responses and Fillet Quality of Rainbow Trout (*Oncorhynchus mykiss*) to Increasing Addition Levels of Dietary Supplementation of Guanidinoacetic Acid

**DOI:** 10.3390/ani15020267

**Published:** 2025-01-18

**Authors:** Pedro Henrique Sessegolo Ferzola, Judith Ringel, Elena Beneder, Carsten Schulz, Martin Gierus

**Affiliations:** 1 Institute of Animal Nutrition, Livestock Products, and Nutrition Physiology, Department of Agrobiotechnology, BOKU University, 1190 Vienna, Austria; pedro.ferzola@boku.ac.at; 2 Austrian Competence Centre for Feed and Food Quality, Safety & Innovation (FFoQSI) GmbH, 3430 Tulln, Austria; 3 AlzChem Trostberg GmbH, 83308 Trostberg, Germany; judith.ringel@alzchem.com; 4 Biotechnological Processes, University of Applied Sciences Wiener Neustadt, Biotech Campus Tulln, 3430 Tulln, Austria; elena.beneder@gmx.at; 5 Institute of Animal Breeding and Husbandry, Department of Marine Aquaculture, Christian-Albrechts Universität zu Kiel, 24118 Kiel, Germany; cschulz@tierzucht.uni-kiel.de; 6 Fraunhofer Research Institution for Individualized and Cell-Based Medical Engineering, Aquaculture and Aquatic Resources, Hafentorn 3, 25761 Büsum, Germany

**Keywords:** guanidinoacetic acid, specific growth rate, fillet quality, rainbow trout

## Abstract

Guanidinoacetic acid (GAA) plays an important role in cellular energy use and protein synthesis. The objectives of this study were to determine the optimal level of dietary GAA regarding the growth performance and fillet characteristics of rainbow trout (*Oncorhynchus mykiss*). A total of 300 trout (initial weight, 66.84 ± 7.82 g) were fed isonitrogenous (34%) and isocaloric (20.6 MJ kg^−1^) diets with increasing levels of GAA (0.00, 0.06, 0.12, 0.18%) for 90 days. The results showed that trout fed GAA (either 0.06, 0.12, or 0.18%) yielded better (*p* < 0.05) performance (BW, BWG, FCR, and SGR) than trout fed the control diet (0.00% GAA inclusion). No differences (*p* > 0.05) were observed for BW, BWG, nor FCR among trout fed GAA. Nonetheless, trout fed a diet with a 0.12% GAA inclusion had the highest (*p* < 0.01) SGR. Fillet quality was not affected by GAA supplementation (*p* > 0.05) for cook loss, shear force, nor colour. Trout fed 0.06% GAA inclusion tended (*p* = 0.08) to have a higher WHC. Moreover, trout fed 0.12 and 0.18% GAA had lower (*p* < 0.05) pH values than trout fed the control diet and 0.06% GAA. Our findings give insights for the growth promotion and fillet quality of trout fed diets supplemented with GAA, especially at the level of 0.12%.

## Simple Summary

The use of feed additives has been increasing in the aquaculture feed industry due to their potential to enhance production and profitability through physiological benefits, such as increased growth rates and fillet quality. Guanidinoacetic acid is a promising feed additive due to its physiological role as creatine precursor and its heat stability during feed manufacturing. The current study was designed to evaluate the effect of increasing doses of guanidinoacetic acid (0.00, 0.06, 0.12, and 0.18%) in rainbow trout (*Oncorhynchus mykiss*) growth, weight gain, and meat quality. We hypothesised that trout fed with the feed additive would have better performance, reaching its optimal physiological response at 0.12%. The results showed that trout fed guanidinoacetic acid presented better weight gain than trout 0.00%, with an optimal growth rate when fed 0.12%. Nonetheless, the level used was not enough to affect fillet quality. This study contributes to the aquaculture industry by determining the optimal level of guanidinoacetic acid for trout performance and its effects on fillet quality. These results can later be adapted to the reality of the aquaculture feed industry to improve fish performance and, consequently, profitability.

## 1. Introduction

Guanidinoacetic acid (GAA), also known as glycoamine or guanidino acetate, is endogenously synthesised from arginine (Arg) and glycine (Gly) in mammals, birds, and fish. When the GAMT enzyme catalyses the methylation between GAA and S-Adenosyl-Homocysteine (SAH), creatine is released and transported to target cells in muscles, brain, and testes. Once in these cells, creatine is phosphorylated by creatine kinase to produce phosphocreatine (PCr), which will store high-energy phosphate to replenish ATP from ADP at times of ATP depletion. Creatine, also known as α-methyl guanidine acetic acid (Cr), has two important functions in animal nutrition and physiology for all animal species. First, it is the precursor of PCr in muscle protein metabolism, which carries high-energy phosphate molecules from the mitochondria to the myosin filaments [1,2]. Second, it acts as a reservoir of high-energy phosphate, regenerating adenosine triphosphate (ATP) from adenosine diphosphate (ADP). PCr acts as a carrier, transporting high energy ATP from the mitochondria to various ATPase sites in the cytosol, especially in tissues of high-energy needs, such as brain and muscles [3,4].

To show the mode of action of GAA, mostly mammals were used. Studies using immortalised mouse myoblast cell lines (C2C12) showed that GAA stimulates myogenic differentiation 1 (MyoD), which is a myogenic regulatory factor (MRF) that induces myogenesis when transfected into non-myogenic cell lines [5]. Also, a study showed that GAA promotes myogenin (MyoG) mRNA expression, increasing the myotube fusion rate [5]. Additionally, GAA supplementation has promoted myotube growth in birds, through an increase in total myosin heavy chain (MyHC) protein levels, myotube thickness, and the gastrocnemius muscle cross-sectional area [6]. GAA also delays the accumulation of lactic acid produced by glycolysis [4], which improves the water-holding capacity (WHC) and muscle tenderness [7,8].

Creatine/phosphocreatine is the main energy buffer in birds and mammals. However, there is a lack of information regarding fish nutrition and how the creatine metabolism could improve energy use [9]. Although physiological responses to GAA supplementation can be transferred from mammals and poultry to fish, some key points must be considered. For instance, contrarily to mammals, Cr in fish might be produced directly in the muscles [4,10,11]. This difference is related to the spatial separation of the steps of Cr synthesis and its consumption. In mammals, GAA is produced in the kidney, converted to Cr by GAMT in the liver, and then transported to the tissues via specific transporters [4]. Meanwhile, in fish the highest expression of enzymes involved in Cr synthesis and the expression of Cr kinases were found directly in the muscle. This results in a more efficient use of Cr, as it is energetically beneficial to maintain the compound directly at the place of its use instead of transporting it through various organs [12]. Additionally, fish muscles have higher Cr contents than mammals [13], which is expected due to the higher proportion of muscle per life weight, but this also leads to a higher Cr turnover.

Cr supplements have been used as growth performance-enhancing agents in livestock because of their pivotal role in energy and muscle metabolism [4]. Nevertheless, Cr has a high supplemental cost [14], instability during manufacturing [15], and relatively low bioavailability [16]. GAA supplementation, on the other hand, has proven to be a stable feed additive in animal feed. GAA’s use as a feed additive increased in the poultry industry after the ban on meat and bone meal in the European market. As Cr is supplied only by animal by-products, the prohibition led to a reduction in poultry performance [6]. In addition, most growing-finishing pig diets in Europe are plant-based, with low contents of Cr [17]. Therefore, as GAA is the precursor of Cr, and its supplementation has been explored to enhance growth performance in the poultry and swine industries as an alternative. In fish, knowledge of the effects of GAA dietary supplementation is still scarce and the recommended dietary inclusion levels are fluctuating. For instance, a study evaluating the effect of four different inclusion levels of GAA (0.00; 0.06; 0.12; and 0.18%) as a feed additive for Nile tilapia (*Oreochromis niloticus*) observed that GAA inclusion, especially at 0.18%, markedly improved the weight gain, serum biochemical parameters, and indicated potential roles as an antioxidant and anti-inflammatory agent [9]. Similarly, tilapia fed diets supplemented with GAA at 0.06 and 0.12% had higher digestibility of protein, Met, Lys, and His in comparison to fish fed diets without GAA [18]. However, carnivore species (e.g., trout (*Oncorhynchus mykiss*)) require the use of animal-based products in feed formulation, as those ingredients serve as Cr source. In contrast, red drum (*Sciaenops ocellatus*) performance when fed diets with 29% Menhanden fishmeal and 2% Cr showed that even with a high inclusion fishmeal in the diet, Cr still has a growth promoting effect [19].

Rainbow trout are an ideal fish species for feeding trials due to their muscle development, economic importance, robustness, and well-established role as a standard species in aquaculture research as its results can be transferred to other salmonid species. The use of feed additives is common in different animal production industries, and it has been of growing interest in the aquaculture sector as well [20]. The present study focused on evaluating the effects of increasing levels of dietary GAA on the performance and the meat (fillet) quality of rainbow trout. We hypothesised that the supplementation of GAA as a feed additive would improve trout body weight and body weight gain, with high feed efficiency. Regarding meat quality, we hypothesised that the addition of 0.12% of dietary GAA would improve trout meat quality parameters, more specifically by increasing fillet WHC, which could lead to the better texture, juiciness, and shelf-life of the food products.

## 2. Materials and Methods

The current trial was approved by the Schleswig-Holstein ethics committee (V 42-7-2023) and the animal ethics committee of BOKU University (BOKU-2023/021). The trial was conducted at the Fraunhofer IMTE aquaculture facility in Büsum, Germany. Rainbow trout (*Oncorhynchus mykiss*) were obtained from a commercial fish farm (Forellenzucht Trostadt, Germany) and transferred to the research facilities in Büsum. Afterwards, fish were stocked randomly into a recirculating aquaculture system (RAS) consisting of 12 rectangular tanks with 150 L water capacity. Water treatment consisted of a mechanical and biological filter and a disinfection unit (UV filter). The photoperiod was set at a 16 L: 8 D cycle. Trout were adapted to the environmental conditions for 4 weeks and were fed with commercial dry feed (Aller Aqua Gold, 3 mm, Aller Aqua, Golssen, Germany). After acclimatisation, the 300 fish were weighed individually, and 25 trout with initial weight of 66.8 ± 7.8 g (mean ± standard deviation) were placed into each of the tanks. Only female trout were used in this trial, as this corresponds to regular production. In male trout, sexual maturity sets in earlier, which leads to growth losses from this point on. In total, 12 tanks were divided in four feeding groups with three replicates (tanks) each. The number of fish per tank is adapted to a minimum stocking density of 10 kg/m^3^ in the individual tanks in order to prevent the aggression that occurs at lower stocking densities.

Trout were fed once a day at 8:30 am until apparent satiation during 90 consecutive days. Satiation was defined as the moment when the fish no longer shows interest on the applied feed. The tanks were cleaned and the water parameters were checked daily (16.5 ± 0.6 °C temperature; 7.7 ± 0.2 pH; 4.61 ± 0.9 ppt salinity, HI 96822 Seawater Refractometer, Hanna Instruments Inc., Woonsocket-RI-USA; 10.46 ± 0.3 mg L^−1^ O_2,_ Handy Polaris, OxyGuard International A/S, Birkerod, Denmark; 0.3 ± 0.2 mg L^−1^ NH_4_-N, 0.9 ± 0.5 mg L^−1^ NO_2_-N, Microquant test kit for NH_4_ and NO_2_; Merck KGaA, Darmstadt, Germany). In total, four diets were formulated (90% DM, 34% CP, and 21 MJ kg^−1^) with increasing levels of GAA inclusion (0.00; 0.06; 0.12; and 0.18%), following the values used in the literature for other fish species [18]. Diets are described in Table 1. All diet ingredients were conditioned and mixed (100 L mixer, 6.0 min conditioning time, 95 °C mixing temperature, 4.6 kg of water addition, and 2.8 kg/h of steam addition). Experimental diets were extruded (OEE 8, Amandus Kahl, Reinbek, Germany) using a 4 mm diameter matrix, ~60 kWh/t, 1:2.5 press ratio, 51 Hz extruder frequency, 84 bar hydraulic pressure, 7.0 kW of performance, throughput of 110.0 kg/h, ~125 °C temperature on extruder head, and ~84 °C after extruder. Animal performance (BW, BWG, FI, and FCR) was assessed every 14 d and at the end of the trial, two fish per tank were randomly sampled to determine body condition indices and nutritional composition. Moreover, another two fish per tank were randomly selected to collect skin and boneless fillet samples to evaluate the sensory quality parameters (colour, pH, CL, SF, and WHC).

### Statistical Analyses

Each water tank was considered as one experimental unit, and the experiment design was a completely randomised. Data were analysed with statistical methods already described in the literature for animal experimentation using the software R Studio (2022.12.0+353, R Development Core Team). After the test for normal distribution (Kolmogorov–Smirnov test) and homogeneity of variances (Levene’s test) [21,22], an analysis of variance (ANOVA) and a parametric post hoc test (Tukey’s HSD test) were carried out [23,24]. Values for each feeding group were presented as the least square means (LS means) and standard error of the mean (SEM). Differences were assumed as significant at *p* ≤ 0.05. Additionally, regression analyses were conducted to determine the optimal inclusion level of GAA in the diet based on animal performance parameters [25].

## 3. Results

**Figure 1:**
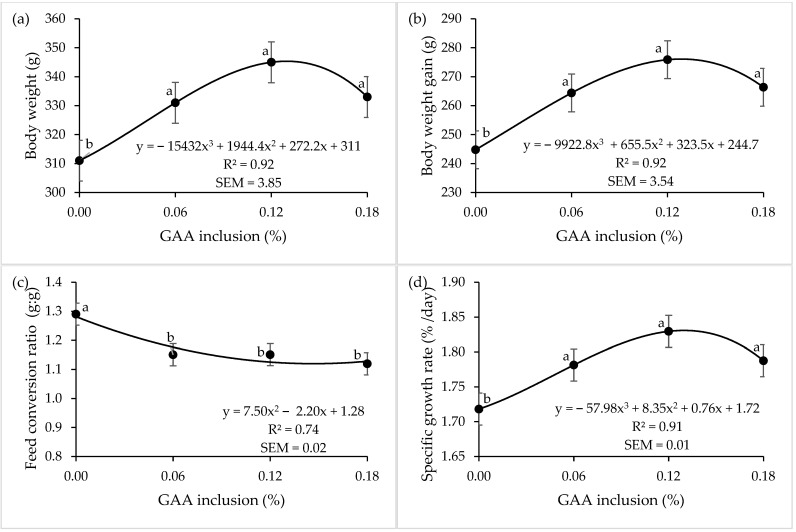
The effects of increasing addition levels of dietary guanidinoacetic acid on rainbow trout performance at 90 d. (**a**) Body weight, (**b**) body weight gain, (**c**) feed conversion ratio, and (**d**) specific growth rate. ^a,b^ Values within a curve not sharing a common superscript differ regarding the effect of increasing addition levels of GAA (*p* ≤ 0.05) by Tukey.

Trout fed with GAA, regardless of the inclusion level, presented a better BW, BWG, FCR, and SGR than trout fed the control diet (0.00% GAA inclusion). No differences among trout fed 0.06, 0.12, and 0.18% of GAA in the diet were observed for BW, BWG, and FCR (*p* > 0.05). Trout fed with 0.06 and 0.18% of GAA had a higher SGR (*p* < 0.01) than fish fed the control diet (0.00% GAA inclusion). Nonetheless, trout fed 0.12% had SGR 0.04% d^−1^ higher (*p* < 0.01) than trout fed 0.06 and 0.18% GAA. Feed intake (FI) was not affected (*p* > 0.05) by GAA inclusion; nevertheless, trout fed 0.12% of GAA showed a tendency (*p* = 0.07) for a higher FI than the other tested groups, which is directly related to the higher BWG and SGR (Table 2). Based on the regressions presented on Figure 1, it is possible to observe that among the inclusion levels used, trout fed 0.12% of GAA presented the highest levels of BW (R^2^ = 0.91), BWG (R^2^ = 0.92), and SGR (R^2^ = 0.91) and the lowest level of FCR (R^2^ = 0.74).

No differences were observed among inclusion levels for the condition factor (*p* > 0.05) nor the viscerosomatic index (*p* > 0.05). However, trout fed the control diet presented a higher hepatosomatic index (*p* = 0.036) than trout fed 0.06% of GAA. Nonetheless, no statistical difference was observed between trout fed the control diet in comparison to 0.12 and 0.18%, as shown in Table 3. Additionally, no differences were observed among the inclusion levels for protein retention (*p* > 0.05) nor energy retention (*p* > 0.05), Table 3.

Regarding fillet quality (Table 4), no statistical differences were observed for cook loss, shear force, nor colour (*p* > 0.05). Nevertheless, trout fed 0.06% GAA presented a tendency (*p* = 0.08) for higher water holding capacity; meanwhile, trout fed control, 0.12, and 0.18% GAA inclusion did not present any statistical differences. Additionally, pH values for trout fed 0.12% were 0.13 and 0.11 lower (*p* < 0.01) than trout fed the control diet and 0.06% GAA, respectively. Trout fed 0.12% GAA also presented higher values of whiteness (L) than trout fed the control diet, but they did not differ statistically from trout fed 0.06 and 0.18% GAA.

## 4. Discussion

Different studies evaluating the effects of GAA inclusion in fish diets sought its results in plant-based diets or in association of low levels of fish meal (FM) inclusion in the diet [18,26,27]. This is because Cr can be obtained only from animal-based protein sources [28], whereas the use of a plant-based diets has a negligible Cr content. In the current study, we evaluated the effects of increasing levels of GAA inclusion in a diet containing 29% FM. FM is an essential ingredient due to its high protein content, amino acid and fatty acid profile, and Cr and GAA content [29,30,31,32]. FM contains 2.0 ± 1 mg/kg of GAA and 1110.5 ± 808 mg/kg of Cr [31], which would already nutritionally benefit fish fed high-FM-diets. Nevertheless, authors seek the replacement of FM in fish feed for its nutritional variations and environmental impact [29,32]. The replacement of FM in fish feed, especially in carnivore species, must be performed with caution, as it may lead to deficiencies of nutrients that can only be supplied by animal protein feedstuffs, such as Cr.

In the current study, we chose to evaluate the effects of GAA supplementation in rainbow trout fed a high-FM diet to assess the nutritional contribution of FM alone and the effects resulting from exogenous GAA. In this scenario, trout fed with GAA improved BW, BWG, and FCR in comparison to the control diet (0.00% GAA inclusion). These findings are supported by the literature, in which studies showed zootechnical improvements in fish fed diets with GAA inclusion levels varying from 0.04 to 0.18% [9,18,26,33]. Nonetheless, high inclusion of FM in the diet does not necessarily mean a high supply of Cr and amino acids. In a previous study, the authors reported a reduction of 10% in the digestibility of CP of fishmeal produced under high thermal treatments, and a reduction in Arg and His digestibility as well [34]. As Cr is a heat sensitive compound, it can be expected that its concentration will be reduced during FM production. In this regard, the use of GAA as a feed additive warrants further investigation, particularly with regard to its nutrient sparing effects and cellular energy buffering to compensate for nutritient losses due to ingredients and feed processing.

The improvement in zootechnical performance with GAA supplementation can be directly attributed to enhanced Cr availability. Cr acts as an intracellular signal and an increase in its synthesis leads to additional energy for cellular bioenergetics and protein synthesis, therefore, resulting in higher fish growth [27,35,36,37]. Furthermore, Cr synthesis in birds and mammals accounts for 40% of the methyl groups of S-adenosylmethionine and uses up to 30% of the amidino groups of Arg [38]. As GAA is endogenously synthesised from Arg and Gly, a spare effect is expected on these amino acids when GAA is exogenously supplied, thereby providing more Arg and Gly available for body protein or endogenous amino acids synthesis [9,15], which may apply for fish too. Additionally, exogenous GAA inclusion may have a secondary effect on the digestibility improvement of protein, Met, Lys, and His digestibility [18]. As GAA is the immediate precursor of Cr and PCr (a high-energy phosphate reserve) its supplementation could promote muscle anabolism by increasing the immediate energy reserves in muscles (ATP and PCr) and increase cellular methylation capacity to support the muscle anabolism [18,39,40], which supports the effects of fish performance in the present study.

No statistical differences were observed among trout fed 0.06, 0.12, and 0.18% GAA inclusion for BW, BWG, and FCR in the present study. Nevertheless, trout fed diet with 0.12% GAA inclusion presented a higher SGR (*p* < 0.01) than the other treatments. Additionally, we observed a slight worsening in the performance of trout fed 0.18% GAA inclusion through the regression analyses (Figure 1). This can be explained by the higher demand of energy for converting Cr into creatinine to eliminate excessive Cr rather than for protein deposition. Similarly, authors reported a reduction in the protein efficiency ratio in tilapia fed 0.12 and 0.18% GAA in comparison to fish fed 0.00 and 0.06% [9]. This effect might be explained by a physiological limit of fish in utilising the dietary protein, and once this optimum level is reached, the excessive protein can be deaminated and catabolized to provide energy to the body. In this step, the protein efficiency is reduced, and the physiological energy expenditure affects animal performance [9].

Compared to poultry or pigs, using other parameters to indicate fish biological status are common. In this regard, no effects were observed in the condition of the trout fed GAA supplementation. The condition factor—a measure of weight per unit length (g/cm^3^)—is often used as an indicator of health, growth, and feeding intensity in fish biology [18]. Additionally, tilapia fed 0.06 and 0.12% GAA had a higher width increment than tilapia fed 0.18% [18], which could lead to their higher acceptability in the market as customers seek for fish with larger dorsal muscles due to a perception of higher quality of the product. In this study, however, no differences were observed for condition factor. The condition factor is also often used as an indirect method to determine the feeding activity. We observed that trout fed 0.12% GAA inclusion tended to have a higher FI (*p* = 0.07) in comparison to the other groups. Nonetheless, no differences were observed for FCR (*p* > 0.05) among fish fed GAA inclusion, but an improvement was observed when compared to the control diet. It can be understood that fish fed GAA inclusion had a higher BW and BWG, which led to a higher FI as the energy required for the body maintenance increased. Although GAA inclusion improved fish zootechnical parameters, the animal growth maintained a proportionality between weight and length, which was translated to the constancy in the condition factor.

Along with the effects on muscle development, Cr has a hydration effect on the muscle cells, drawing water into them, which promotes protein synthesis, reduces proteolysis, and enhances glycogen synthesis [36]. GAA, as a Cr precursor, may affect sensory characteristics of muscle (fillet) quality. We observed a lower (*p* < 0.01) pH in the fillets from fish fed diets 0.12 and 0.18% GAA inclusion in comparison to fish fed the control and 0.06% GAA inclusion diets, which might occur due to a higher concentration of Cr phosphate in the muscle [41]. A lower pH can be correlated to a lower fillet quality [42]. Nevertheless, our results are in the range reported for trout in the literature. Authors reported trout fillet with pH values varying from 6.1 to 6.9 depending on storage time [43]. A trial reported with Grass carp (*Ctenopharygodon idella*) fed diets with up to 0.06% GAA inclusion, in which the authors conducted a trial replacing 100% of FM used in the diet by a vegetable meal with increasing levels of GAA inclusion [27]. The authors reported that fish fed a diet with FM had a pH of 6.39 and fish fed a diet with vegetable meal and 0.06% GAA inclusion showed a similar pH of 6.30. On the one hand, these values corroborate the ones observed in the current trial for diets with 0.00 (6.38) and 0.06% (6.36) GAA inclusion, considering that our diets had a high inclusion of FM of 29%. On the other hand, the reduction on the pH values on the diets with 0.12 and 0.18% might be a response to the oxidant–antioxidant mechanism facilitated by GAA supplementation. It has been reported that GAA may act as antioxidant agent, as well as pro-oxidant agent, resulting in an over production of reactive oxygen species (ROS) and leading to oxidative stress [44]. Muscle pH is also directly connected to WHC, in which high pH values tend to increase WHC. In this trial, we did not observe a statistical difference for WHC among treatments, but a tendency (*p* = 0.07) where fish were fed a diet of 0.06% GAA inclusion had WHC values ~2.3% higher than the others. Due to the higher inclusion of fish meal in feeds, the GAA supply at different levels was not enough to alter the fillet quality.

## 5. Conclusions

Our findings revealed that even with high inclusion of fish meal in the diet, the supplementation of guanidinoacetic acid (GAA) as a feed additive improved trout zootechnical performance. The effects of exogenous GAA were attributed to the sparing effect of amino acids (especially Arg and Gly) and to the higher creatine availability. Our results indicated that a 0.12% GAA inclusion level optimised the specific growth rate. Additionally, the regression lines showed that 0.12% of GAA inclusion in the diet was the optimum level for rainbow trout zootechnical performance (body weight gain, feed conversion ratio, and specific growth rate). Nonetheless, GAA supplementation with the levels used in this study was not enough to alter the fillet quality, probably due to the creatine availability from fish meal.

Based on the effects of GAA on rainbow trout zootechnical performance, GAA seems to be an interesting feed additive to be used in the fish feed industry. The authors did not focus on the cost–benefits of GAA in fish feed, but on its effects on zootechnical performance and fillet quality instead. On this regard, future studies should evaluate the economic impact of GAA as a feed additive when fish are fed different supplementation levels of GAA in association with different inclusion levels of fish meal in the diet.

## Figures and Tables

**Table 1:** Formulation (g kg^−1^ feed), proximate composition (g kg^−1^ dry matter), and amino acid composition (g kg^−1^ crude protein) of experimental diets.

	GAA 1	GAA 2	GAA 3	GAA 4
*Ingredients*				
Soybean meal 48%	367.7	367.1	366.5	365.9
Fish meal 65%	292.3	292.3	292.3	292.3
Wheat bran 15%	190.0	190.0	190.0	190.0
Oil	140.0	140.0	140.0	140.0
Vit./Min. mix ^1^	10.0	10.0	10.0	10.0
Guanidinoacetic acid	0.0	0.6	1.2	1.8
*Proximate composition*				
Dry matter	903.3	903.7	903.2	902.7
Crude protein	348.7	344.4	338.2	345.2
Ether extract ^2^	104.3	103.0	101.1	103.2
Crude fibre	28.9	28.6	28.0	28.6
Gross energy (MJ kg^−1^)	21.0	20.7	20.5	20.5
Ash	89.3	87.0	86.2	86.0
Ca	16.0	16.6	15.9	15.5
P	7.9	6.9	7.7	7.8
*Essential amino acids*				
Arginine	37.7	37.2	36.6	37.3
Histidine	15.9	15.8	15.5	15.8
Isoleucine	25.4	25.1	24.6	25.1
Leucine	44.2	43.7	42.9	43.8
Lysine	44.9	44.4	43.6	44.5
Methionine	24.7	24.4	24.0	24.5
Phenylalanine	24.7	24.4	23.9	24.5
Methionine + Cysteine	30.6	30.2	29.7	30.3
Threonine	2.5	2.4	2.4	2.5
Valine	30.5	30.1	29.5	30.1
Glycine + Serine	6.1	6.0	5.9	6.0

^1^ Vitamin and mineral premix nutritional composition: vitamin A (3a672a) 800,000, i.e., vitamin D3 (3a671) 500,000 I.E.; betaine (betaine anhydrate 3a920) 84,000 mg; choline chloride (3a890) 80,000 mg; vitamin E all-racalpha-tocopheryl acetate (3a700) 40,000 mg; vitamin C (L ascorbic acid 3a300) 30,000 mg; niacinamide (3a315) 30,000 mg; calcium D- pantothenate (3a841) 10,000 mg; vitamin B2 (3a825) 4000 mg; vitamin B6 (3a831) 3000 mg; vitamin B1 (3a821) 3000 mg; vitamin K3 (3a710) 2000 mg; folic acid (3a316) 1,600,000 μg; biotin (3a880) 160,000 μg; vitamin B12 (3a) 10,000 μg; iron (II) sulphate monohydrate (3b103) 30,000 mg; zinc (zinc oxide 3b603) 12,000 mg; manganese (manganese (II) oxide 3b502) 5000 mg; iodine (calcium iodate, anhydrous 3b202) 800 mg; copper (copper (II) sulphate), pentahydrate (3b405) 600 mg; selenium (sodium selenite 3b801) 40 mg. ^2^ With acid hydrolysis^.^

**Table 2:** The effects of increasing addition levels of dietary guanidinoacetic acid on rainbow trout performance at 90 d.

GAA (%)	BW (g)	BWG (g)	FI (g)	FCR (g:g)	SGR (%d^−1^)
0.00	311 ^b^	245 ^b^	308	1.29 ^a^	1.72 ^c^
0.06	331 ^a^	264 ^a^	304	1.15 ^b^	1.78 ^b^
0.12	342 ^a^	275 ^a^	329	1.15 ^b^	1.82 ^a^
0.18	329 ^a^	262 ^a^	298	1.14 ^b^	1.78 ^b^
S.E.M.	3.21	3.07	7.04	0.018	0.07
	*p*-value				
	<0.01	<0.01	0.07	<0.01	<0.01

^a–c^ Values within a line not sharing a common superscript differ regarding the effect of increasing addition levels of GAA (*p* ≤ 0.05) by Tukey. BW: individual body weight. BWG: individual body weight gain. FI: feed intake. FCR: feed conversion ratio. SGR: specific growth rate (% day) = [ln (W_f_) − ln (W_i_)]/feeding days × 100.

**Table 3:** The effects of increasing addition levels of dietary guanidinoacetic acid on rainbow trout body condition indices at 90 d.

GAA (%)	CF (g/cm^3^)	HSI (%)	VSI (%)	CPR (%)	GER (%)
0.00	1.54	1.75 ^a^	15.8	44.8	44.4
0.06	1.51	1.32 ^b^	14.7	46.0	48.8
0.12	1.50	1.56 ^ab^	14.9	46.2	48.5
0.18	1.45	1.50 ^ab^	14.9	48.0	52.0
S.E.M.	0.038	0.094	0.060	2.16	1.87
	*p*-value				
	>0.05	0.036	>0.05	>0.05	>0.05

^a,b^ Values within a line not sharing a common superscript differ regarding the effect of increasing addition levels of GAA (*p* ≤ 0.05) by Tukey. CF: condition factor (body weight/total length^3^) × 100. HSI: hepatosomatic index (liver weight/body weight) × 100. VSI: viscerosomatic index (visceral weight/body weight) × 100. CPR: crude protein retention. GER: gross energy retention.

**Table 4:** The effects of increasing addition levels of dietary guanidinoacetic acid on rainbow trout fillet quality at 90 d.

GAA (%)	WHC (%)	CL (%)	pH	SF (kg)	Whiteness	Chroma
0.00	57.9 ^b^	13.7	6.38 ^a^	1.31	51.4 ^b^	18.4
0.06	60.4 ^a^	12.5	6.36 ^a^	1.37	52.3 ^ab^	20.4
0.12	58.7 ^b^	12.0	6.25 ^b^	1.36	53.2 ^a^	19.5
0.18	57.7 ^b^	13.3	6.28 ^b^	1.35	52.6 ^ab^	18.6
S.E.M.	0.85	0.039	0.023	0.10	0.36	0.88
	*p*-value					
	0.08	>0.05	<0.01	>0.05	<0.05	>0.05

^a,b^ Values within a line not sharing a common superscript differ regarding the effect of increasing addition levels of GAA (*p* ≤ 0.05) by Tukey. WHC: water-holding capacity. CL: cook loss. SF: shear force. Whiteness: 100 − [(100 − L∗)^2^ + a∗^2^ + b∗^2^]^1/2^. Chroma: chromatography = a × 2 + b × 2

## Data Availability

Data are contained within the article.
